# Poly-Ingredient Formulation Bresol^®^ Ameliorates Experimental Chronic Obstructive Pulmonary Disease (COPD) in Rats

**DOI:** 10.3797/scipharm.1212-06

**Published:** 2013-05-09

**Authors:** Mohamed Rafiq, Gollapalle Lakshminarayanashastry Viswanatha, Dattatray Anturlikar Suryakanth, Mohammed Azeemuddin, Mahalingaiah Jagadeesh, Krishna Dhanush, Pralhad Sadashiv Patki

**Affiliations:** Department of Pharmacology, R&D Center, The Himalaya Drug Company, Makali, Bangalore, Karnataka, India.

**Keywords:** Bresol^®^, Cigarette smoke-induced lung injury, Bronchitis, Broncheoalveolar lavage fluid, TNF-α

## Abstract

In the present study, the protective effect of Bresol^®^ – a polyherbal formulation – was evaluated in an experimental model of cigarette smoke (CS)-induced COPD in rats. Ten minutes daily exposure to CS for 7 weeks caused significant elevation of TNF-α (p<0.01) and total protein (p<0.01) in the bronchoalveolar lavage fluid (BALF) of positive untreated control animals, indicating ongoing inflammatory process in the lungs. Further, histopathological findings have confirmed the presence of pathological lesions in the trachea and lungs. Five weeks of post-treatment with Bresol^®^ (250 and 500 mg/kg, p.o.) showed significant and dose-dependent anti-inflammatory effects against CS-induced lung abnormalities by maintaining the TNF-α and total protein levels within the normal range. Additionally, Bresol^®^-treated animals showed normal cyto-architecture of the trachea and lungs. In conclusion, Bresol^®^ showed dose-dependent protection against CS-induced lung and tracheal injury in rats, which further indicates, Bresol^®^ is a useful healing agent, may help to decelerate the progression of COPD, and reduce the exacerbations in patients.

## Introduction

Chronic obstructive pulmonary disease (COPD) is a heterogeneous group of airway inflammatory diseases, which is associated with chronic bronchitis, emphysema, and small airway remodeling. It is slowly progressive and largely irreversible, characterized by airway obstruction, cough, increased sputum secretion, and shortness of breath *etc*[1, 2].

The most important risk factor for COPD includes cigarette smoking, exposures to dust, fumes, polluted air particles, and biomass fuels etc. Genetic predisposition also seems to play an important role in the development of COPD [3]. According to WHO, it is the fifth-most common cause of morbidity and the third-most common chronic disease worldwide. Thus, COPD will be one of the most important health care problems in the near future [4].

The pharmacotherapy of COPD mainly includes the use of bronchodilators, corticosteroids, mucolytics, antioxidants, smoking cessation drugs, antibiotics, and immune-regulators etc. Even though varieties of agents are prescribed, the outcome of treatment is variable and unsatisfactory [3, 5, 6].

Bresol^®^ is a poly-ingredient formulation approved by the Government of India’s Drug Regulatory Authority (Department of Ayush, Ministry of Health and Family Welfare); it has been available in the market for several years. The composition of Bresol^®^ with respect to the scientific names of the plants, parts used, type of herbal preparation, and amount of each herbal preparation in the formula is given in Table 1.

Previously, Bresol^®^ demonstrated its therapeutic potential in the treatment of allergic conditions, and pharmacological investigations suggest its action on different pathways leading to hypersensitivity and inflammation [7–9].

The cigarette smoke (CS)-induced rat model of COPD exhibited various structural changes such as airspace enlargement in the lungs or emphysema, inflammatory changes and hyperplasia of mucus-secreting goblet cells in the airway tissues, apart from other biochemical changes which resemble the clinical features of COPD in patients. This experimental model is widely reported and used to evaluate the drugs which may be useful in COPD.

With this background, the present study was undertaken to investigate the beneficial effect of Bresol^®^ in an experimental model of CS-induced chronic obstructive airways disease (COPD) in rats.

## Results

### Cigarette Smoke-induced COPD

In the present study, seven weeks of chronic exposure to cigarette smoke caused damage to the histoarchitecture of the trachea and lungs of Wistar rats, which was observed by means elevated cytokine levels (TNF-α) and total protein concentrations in the BALF. In addition, histopathology reports confirmed the bronchitis caused by the cigarette smoke.

### Protein Estimation in BALF

**Fig. 1:**
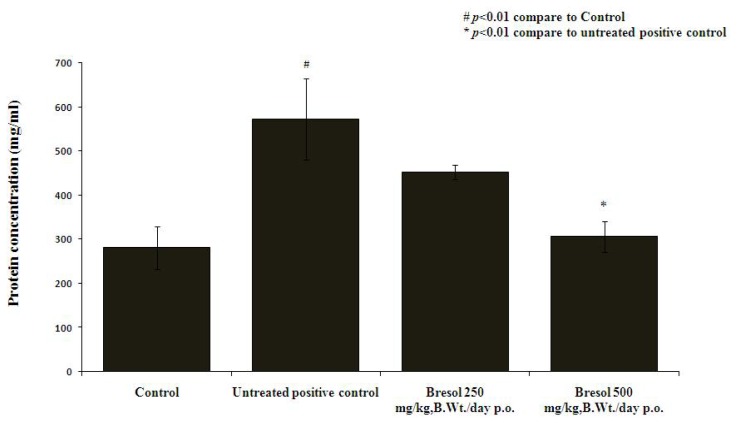
Effect of Bresol on CS-induced elevated total protein levels in BALF. Values are expressed as mean ± SEM (n=6); all the groups were statistically compared by ANOVA followed by Tukey’s multiple comparison. ^#^*p*<0.01 compare to control, *p<0.01 compare to positive control.

The untreated positive control group showed a significant elevation of total protein concentrations in the BALF compared to the normal control (p<0.05). Further, the Bresol^®^ (500 mg/kg)-treated group exhibited total protein concentration almost near to the normal control (p<0.01), while the lower dose of Bresol^®^ (250 mg/kg) did not significantly reduce the CS-induced elevation of total protein levels in the BALF (Fig. 1).

### TNF-alpha Concentration in BALF

**Fig. 2:**
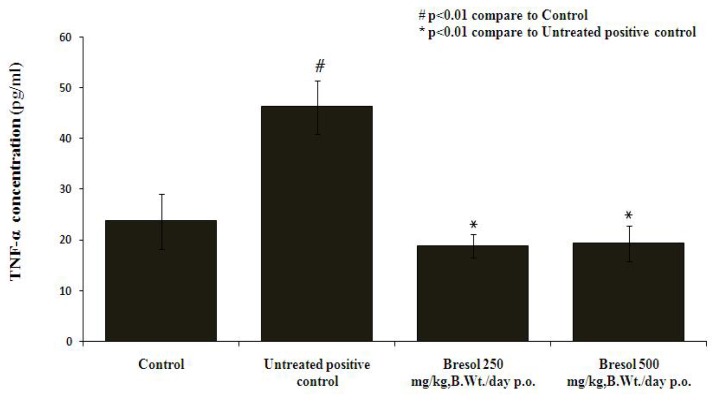
Effect of Bresol on CS- induced elevated TNF-α levels in BALF. Values are expressed as mean ± SEM (n=6); all the groups were statistically compared by ANOVA followed by Tukey’s multiple comparison. ^##^*p*<0.01 compare to control, *p<0.01 compare to positive control.

In line with the protein estimation, the untreated positive control group showed a significant elevation of TNF-α concentrations in the BALF compared to the normal control (p<0.01), indicating induced bronchitis. Bresol^®^ (250 and 500 mg/kg) treatment significantly prevented the elevation of TNF-α levels in the BALF (p<0.01). The results are given in Fig. 2.

### Histopathology

**Fig. 3:**
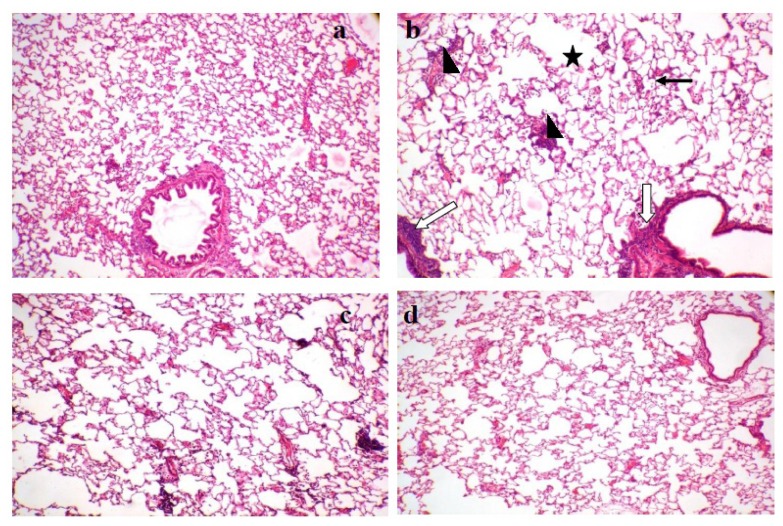
Effect of Bresol on CS-induced altered Histology of Lungs. (a) Lung of control group showing normal architecture, (b) Disease control showing alveolar enlargement/emphysema (asterisk), increased number of macrophage (black arrow), multifocal lymphocyte aggregates (arrowhead) and peribronchial inflammation (white arrow), (c) Bresol-250 mg/kg showing moderate alveolar enlargement/emphysema, mild increase in the number of macrophage and multifocal lymphocyte aggregates, (d) Bresol-500 mg/kg mild alveolar enlargement/emphysema, minimal increase in the number of macrophage and multifocal lymphocyte aggregates and absence of peribronchial inflammation. (Hematoxylin (H) and Eosin (E) staining, 100 × Magnification).

**Fig. 4:**
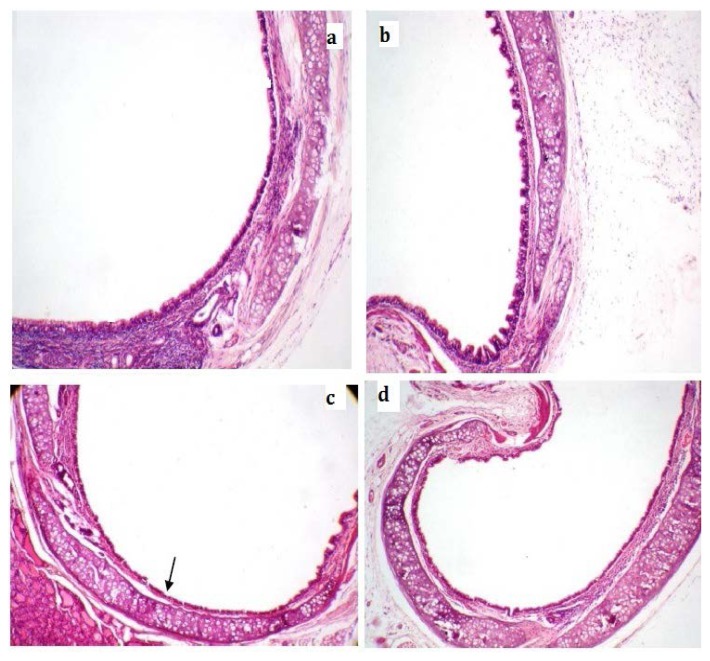
Effect of Bresol on CS-induced altered Histology of Trachea. (a) Trachea of control group showing normal architecture, (b) Disease control showing mild goblet cell hyperplasia, (c) Bresol-250 mg/kg showing minimal to mild goblet cell hyperplasia, (d) Bresol-500 mg/kg showing almost normal architecture of trachea (Hematoxylin (H) and Eosin (E) staining, 100 × Magnification).

The control animals showed normal cytoartitechture of the lungs and trachea. In contrast, the untreated positive control animals showed alveolar enlargement/emphysema; increase in the number of macrophages, multifocal lymphocyte aggregates, and an associated with peribronchial inflammation and mild goblet cell hyperplasia in the trachea. Bresol^®^ 250 and 500 mg/kg treatments alleviated the CS-induced bronchitis; the histopathology of the lungs showed moderate alveolar enlargement/emphysema, a mild increase in the number of macrophages, and multifocal lymphocyte aggregates, but there was no observed peribronchial inflammation in the treated groups. The histopathology of the trachea showed normal architecture in the Bresol^®^ (500mg/kg, p.o.) group, while Bresol^®^-250 mg/kg treated group showed minimal to mild goblet cell hyperplasia in the trachea (Fig. 3 & 4).

## Discussion

Cigarette smoke (CS) plays a key role in the development of COPD [13]. The presence of more than 4,700 chemical compounds, including high concentrations of free radical and other oxidants in CS, are thought to be responsible for inducing an imbalance between oxidants and antioxidants, which is identified as a major etiological factor in the pathogenesis of COPD [14].

Smoking causes airway and systemic inflammation. In the early inflammatory response, bronchial and alveolar epithelial cells secrete pro-inflammatory cytokines such as TNF-α and IL-1β to activate alveolar macrophages (AM), which in turn release more cytokines and chemokines (IL-8 in human/CINC-1 in rats), leading to neutrophil recruitment and activation [15].

In the CS exposure models, oxidants and matrix metalloprotenases (MMPs) complement each other in damaging the lung tissue [16]. Together with the generation of about 10^14^ free radicals per puff of CS, endogenous reactive oxygen species are also produced in the lung in normal cellular processes. and malondialdehyde levels have been used as a convenient index of lipid peroxidation–related oxidative damage from smokers [17, 18]. Also, oxidative stress induced by chronic CS exposure caused alveolar and lung damage, associated with elevated levels of total protein and TNF-α, which in turn stimulate the alveolar macrophages to release matrix metalloprotenases (MMP) [19].

In the present study, there was a significant elevation of total protein and TNF-α levels in BALF of the untreated positive control group compared to the normal control. These findings suggest that total protein and TNF-α play a pivotal role in CS-induced bronchitis. The findings of our study are in line with the available literature on CS-induced bronchitis in experimental animals [20, 21]. Similar to our findings, the up-regulation of pro-inflammatory mediators has been found in patients with COPD in different biological samples including blood, sputum, BALF, and exhaled breath condensate [20–23]. In support of biochemical estimations, the histopathology of the untreated positive control animals showed characteristics of severe respiratory damage. Treatment of the animals with Bresol^®^ 250 and 500 mg/kg, p.o. ameliorated the biochemical and histological alterations observed due to chronic CS exposure.

The above observations suggest that Bresol^®^ exhibits anti-inflammatory activity in airways and ameliorates experimental COPD in rats. From the present findings, it can be concluded that Bresol^®^ could be a useful healing agent, which could help to decelerate the progression of COPD and may be a useful in reducing the exacerbations in patients.

## Experimental

### Drugs and Chemicals

The rat TNF-α Elisa kit (Krishgen Biosystems, Mumbai) and Bradford reagent (Sigma-Aldrich, Bangalore) were used; all other chemicals and reagents used were highly pure and analytical grade purchased from local firms.

### Experimental Animals

In-bred male Wistar rats (200–250g) were used for the study; all the animals were housed under standard laboratory conditions, air-conditioned with an adequate fresh air supply (12–15 air changes/hour). Animals were maintained under standard conditions, i.e. room temperature 19–25°C, relative humidity 30–70% and 12:12 h light-dark cycle. Before and during the experimental period, the animals were provided standard rat pellet and water *ad libitum*. The experimental protocols were approved by the Institutional Animal Ethics Committee (IAEC) of The Himalaya Drug Company, Makali, Bangalore and all the experiments were conducted according to the guidelines of the Committee for the Purpose of Control and Supervision on Experiments on Animals (CPCSEA), Ministry of Environment and Forests, Government of India.

### Cigarette Smoke-induced COPD

Male Wistar rats (200–250g) were randomized into four groups (n=10) (G-I to G-IV) based on body weight, and the mean body weight variation between the groups was kept under 10 grams. G-I is the normal control, G-II is the untreated positive control, G-III and G-IV received Bresol^®^ 250 and 500 mg/kg, respectively, and for inducing COPD, all the animals except control group (G1) were exposed to CS daily for 10 mins in a transparent acrylic chamber (2 feet diameter × 1 feet height) connected to vacuum pump (0.4 psi capacity) at one end and at other end a cigarette was fixed, and the entire setup was enclosed in a fume hood. Charminar brand cigarretes were used which contained 31 mg total particulate matter/cigarette and 1.96 mg total nicotine alkaloid/cigarette.

After 15 days of the induction period, G-I and G-II received DM water (10 ml/kg, p.o.), then G-III and G-IV were treated daily with Bresol^®^ 250 and 500 mg/kg, p.o. 2 hours after exposure to cigarette smoke for 5 weeks. At the end of the treatment, all the animals were euthanized and the BALF was collected using buffered saline solution and volume was adjusted. The airway tissue, such as the lungs along with the trachea, was collected for histopathological evaluation [1, 10, 11]. The experimental conclusions made in the manuscript are based on the single experiment conducted.

### Histopathological Evaluation

After the completion of the experiment, all the animals were sacrificed by an overdose of anaesthesia, and the lungs along with the trachea were collected from all the animals, the tissue was infused with 10% neutral buffered formalin (10% NBF) to avoid atelectasis artifacts, and immersed in neutral buffered formalin. Lung tissues were embedded in paraffin, 5-μm sections were obtained, and tissues were processed by routine histological methods and stained with Hematoxylin (H) and Eosin (E), and evaluated microscopically.

### Collection of Bronchoalveolar Fluid (BALF)

Airspaces in the right lung were washed with buffered saline solution (500 μL) successively three times (final volume 1.2–1.5 mL). The fluid was withdrawn and centrifuged at 3000 × *g* for 5 min. The supernatants and sediments were separated and stored at −20°C [1].

### Protein Estimation in BALF

The protein concentration was estimated by Bradford’s method using bovine serum albumin (BSA) as the standard according to the Lowry method [12].

### Enzyme-linked Immunosorbent Assay

Tumor necrosis factor-α (TNF-α) in BALF was quantified with a specific enzyme-linked immunosorbent assay using a rat TNF-α kit according to the manufacturer’s instructions (http://www.krishgen.com/Resources/rTNFa_MSDS_KB3016_E.pdf, Krishgen Biosystems, Mumbai), and rat recombinant TNF-α was used as a standard in this assay.

### Statistical Analysis

All the values were expressed as Mean ± SEM, the results were statistically analyzed by One-way ANOVA followed by Tukey’s post test using using GraphPad Prism version 4.01 for Windows, GraphPad Software, San Diego California USA, and a *p* value less than 0.05 was considered statistically significant.

## Figures and Tables

**Tab. 1:** Composition of Bresol^®^ granules

No.	Ingredients	Part used	Percentage yield of the extract	Proportion of extract used in the granules (%)
1	*Curcuma longa*	Rhizome	12.00	39.80
2	Trikatu*	Rhizome & Fruits	10.00	3.17
3	Triphala#	Fruits	50.00	3.17
4	*Cinnamomum zeylanicum*	Bark	3.00	1.98
5	*Elettaria cardamomum*	Fruit	10.00	1.98
6	*Cinnamomum tamala*	Leaf	9.00	1.98
7	*Embelia ribes*	Fruit	9.00	3.17
8	*Cyperus rotundus*	Tuber	11.00	3.17
9	*Mesua ferrea*	Flower	12.00	1.98
10	*Ocimum sanctum*	Whole Plant	8.00	19.80
11	*Adhatoda vasica*	Leaf	22.03	19.80

Solvent used: water.

* …contains equi-proportion of Zingiber officinale (Rhizomes), Piper longum(Fruit) and Piper nigrum (Fruit).

# …contains equi-proportion of Terminali chebula (Fruit), Terminalia bellerica (Fruit) and Emblica officinalis (Fruit).
